# Discovery of Small Molecule Activators of Chemokine Receptor CXCR4 That Improve Diabetic Wound Healing

**DOI:** 10.3390/ijms23042196

**Published:** 2022-02-16

**Authors:** Junwang Xu, Junyi Hu, Shaquia Idlett-Ali, Liping Zhang, Karly Caples, Satyamaheshwar Peddibhotla, Morgan Reeves, Carlos Zgheib, Siobhan Malany, Kenneth W. Liechty

**Affiliations:** 1 Department of Physiology, University of Tennessee Health Science Center, Memphis, TN 38163, USA; jxu61@uthsc.edu; 2 Laboratory for Fetal and Regenerative Biology, Department of Surgery, Anschutz Medical Campus, University of Colorado Denver Aurora, Denver, CO 80045, USA; junyi.hu@cuanschutz.edu (J.H.); shaquia.idlett-ali@cuanschutz.edu (S.I.-A.); lzhan112@uthsc.edu (L.Z.); carlos.zgheib@ucdenver.edu (C.Z.); 3 Department of Pharmacodynamics, College of Pharmacy, University of Florida, Gainesville, FL 32610, USA; kcaples@cop.ufl.edu (K.C.); speddibhotla@cop.ufl.edu (S.P.); morgan.reeves@ufl.edu (M.R.)

**Keywords:** high-throughput screening, CXCR4 agonists, diabetic wounds, chemical compounds

## Abstract

Diabetes produces a chronic inflammatory state that contributes to the development of vascular disease and impaired wound healing. Despite the known individual and societal impacts of diabetic ulcers, there are limited therapies effective at improving healing. Stromal cell-derived factor 1α (SDF-1α) is a CXC chemokine that functions via activation of the CXC chemokine receptor type 4 (CXCR4) receptor to recruit hematopoietic cells to locations of tissue injury and promote tissue repair. The expression of SDF-1α is reduced in diabetic wounds, suggesting a potential contribution to wound healing impairment and presenting the CXCR4 receptor as a target for therapeutic investigations. We developed a high-throughput β-arrestin recruitment assay and conducted structure–activity relationship (SAR) studies to screen compounds for utility as CXCR4 agonists. We identified CXCR4 agonist UCUF-728 from our studies and further validated its activity in vitro in diabetic fibroblasts. UCUF-728 reduced overexpression of miRNA-15b and miRNA-29a, negative regulators of angiogenesis and type I collagen production, respectively, in diabetic fibroblasts. In vivo, UCUF-728 reduced the wound closure time by 36% and increased the evidence of angiogenesis in diabetic mice. Together, this work demonstrates the clinical potential of small molecule CXCR4 agonists as novel therapies for pathologic wound healing in diabetes.

## 1. Introduction

Diabetes has reached pandemic proportions worldwide. Complications of diabetes, such as impaired wound healing, represent a significant medical problem, with the annual cost of diabetic lower extremity ulcers alone exceeding 1.5 billion dollars [1]. These chronic wounds result in significant morbidity for individuals, including long hospitalizations, prolonged exposure to antibiotics, acute and chronic pain, the need for cumbersome wound care, and restricted mobility. In addition, an ulcer of the lower extremity precedes 84% of all diabetic lower extremity amputations, and is the primary cause for hospitalization among diabetics [2]. Despite the enormous impact of these chronic wounds on both individuals and society, effective therapies are lacking. Thus, the modification, correction, or prevention of diabetes impaired wound healing has far-reaching consequences, both on patient outcomes and on healthcare expenditures.

Normal wound repair follows an orderly and well-defined sequence of events that requires the interaction of many cell types, such as inflammatory cells, fibroblasts, keratinocytes, endothelial cells and progenitor cells, as well as the involvement of many growth factors, extracellular matrix (ECM) proteins, and enzymes. In diabetic wound healing, this complex orchestration of wound healing processes is disrupted. This impairment is associated with the significantly decreased production of granulation tissue and an increased epithelial gap, compared to non-diabetic wounds [3,4,5,6,7]. The production of granulation tissue is dependent on the formation of new vessels in the wound bed, synthesis of extracellular matrix (ECM), and provides the substrate for epithelial cell migration and subsequent wound closure.

The stromal cell-derived factor1α (SDF-1α) is a CXC chemokine, important in the mobilization and recruitment of hematopoietic progenitor cells and other CXCR4+ cells to bone marrow and other tissues [8]. SDF-1α secretion has also been shown to be upregulated by hypoxia inducible factor (HIF-1α) and its expression is increased in areas of tissue injury [9,10]. These observations have led to suggestions that SDF-1α may also have a central role in directing cells to injured tissues to facilitate tissue repair.

On analysis, we found that diabetic wounds produced significantly less SDF-1α both at an mRNA and protein level [3] compared to normal wound tissue. We have previously shown that the local application of Mesenchymal Stem Cells (MSCs) enhances wound closure in a diabetic mouse model [11]. We found that MSC-treated wounds had a decreased inflammation response, decreased epithelial gap, and increased vessel density [11]. Furthermore, this effect appears to be due, in part, to the increased production of SDF-1α in wounds [11].

In a gain of function experiment, we overexpressed SDF-1α by lentivirus vector, and found that SDF1 treatment resulted in greater granulation tissue, a smaller epithelial gap, and a smaller wound size in diabetic wounds [3]. In a loss of function experiment, we injected a lentiviral vector that expresses a mutant form of SDF-1α that binds, but does not activate, CXC chemokine receptor type 4 (CXCR4) and measured its effect on granulation tissue formation, angiogenesis, inflammation, cell migration, and wound healing. We found that competitive inhibition of SDF-1α significantly impairs the rate of wound healing, decreases angiogenesis, and increases inflammation in the diabetic mouse [12].

In summary, previous data indicated that SDF-1α is a key factor in the wound-healing process that could be targeted to correct the diabetic wound-healing defect. Therefore, screening for small molecule positive modulators that can activate the CXCR4 receptor and its downstream signaling pathway, thereby blocking SDF-1α response, which will provide a novel topical therapy for diabetic wound healing with a reduced risk for systemic receptor activation, and has a great potential for clinical application and commercialization.

## 2. Results

### 2.1. Application of the Cell-Based High-Throughput Screening

**Figure 1:**
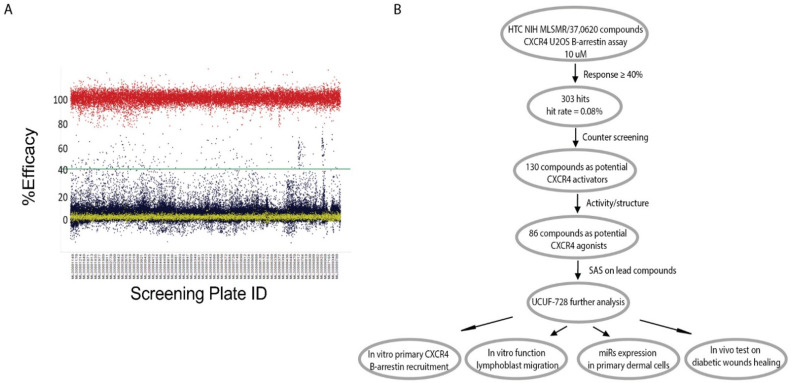
High-Throughput Screening for CXCR4 Activators. (**A**) Scatter plot of test compounds at 10 mM (blue). For positive (high) control (red dots), 100 nM SDF-1α was used, and DMSO was used as negative (low) control (yellow dots). DMSO in all wells was <0.5%. Z′ = 0.83. (**B**) Assay cascade for identification of lead CXCR4 activator and the workflow of this study.

In our effort to identify CXCR4 activators from the NIH Molecular Library Small Molecule Repository (MLSMR), we developed a primary screening assay to measure β-arrestin recruitment to the activated G-protein coupled receptor (GPCR) by a Florescence Resonance Energy Transfer (FRET) in U2OS cells, overexpressing the CXCR4 receptor engineered with a β-lactamase (bla) reporter system. CXCR4-bla cells plated in 1536 w were stimulated in the presence of 10 µM compound concentration. The quantity of arrestin binding was detected by a decrease in a FRET-enabled β-lactamase substrate and normalized to a maximal amount, produced by an EC_80_ concentration of SDF-1α. Using this approach, we successfully screened the NIH MLSMR of 370,620 compounds (Figure 1A,B) and identified 303 hits (hit rate = 0.08%). Hit confirmation resulted in 130 compounds as potential CXCR4 activators (response ≥ 40% activity and filtered for florescence interference in the 535 nm channel). These compounds were graded according to activity and structure, resulting in 86 compounds that met the criteria as potential CXCR4 agonists for further characterization (Figure 1B). The workflow for high-throughput screening and this study is illustrated in Figure 1B.

### 2.2. Identified Lead Compounds by Structure–Activity Studies

Commercially available compounds representing three distinct scaffolds were purchased, and EC_50_ values were determined in the primary 1536 w β-arrestin recruitment assay using SDF-1α from R&D systems. Chemical stability, synthetic tractability, solubility and complete dose response and potency criteria led to the prioritization of the 5-aryl-2-cyclopropyl oxazole scaffold, represented by primary hit Compound 2 (Table 1). The early structure–activity relationship was established around the 5-aryl-2-cyclopropyl oxazole core with commercially available analogs with diverse substituents in the sulfonanilide ring of the scaffold. Electron-rich alkoxy substituents at 2-position were the most promising in terms of activity among the monosubstituted sulfonanilide derivatives, and 2-ethoxy analog had an EC_50_ of 1.0 μM (Table 1 entries 5 vs. 1–7). Moving the alkoxy substituent to 3- and 4-position led to a significant loss of activity, indicating that the 2-alkoxy substituents might lock the anilide ring in a favored conformation (entries 8–9). The screening of several dimethyl substituents (entries 10–15) confirmed the above observation, and of 2,5-dimethyl, 13 and 2,6-dimethyl analogs, 14 were found to be the most active among the dimethyl analogs due to their potential ability to force the anilide ring in a favorable non-planar orientation compared to the rest of the molecule. Keeping the 2-OMe substituent constant, we screened a small set of electron withdrawing and electron rich substituents at the 5-position of the anilide ring (entries 16–19). We observed that electron rich groups are better suited at the 5-position and the 2,5-dimethoxy analog, 19 (UCUF-728), had the best activity and agonist response in the 1536 w screening platform (EC_50_ = 1.1 μM and E_max_ = 60%). The 3,4 and 3,5-dimethoxy analogs were inactive or had marginal activity, further confirming our observations (entries 20 and 21). The analytical data for UCUF-728 is shown in Supplemental Figures S1–S4.

### 2.3. In Vitro Biological Profile of UCUF-728

**Figure 2:**
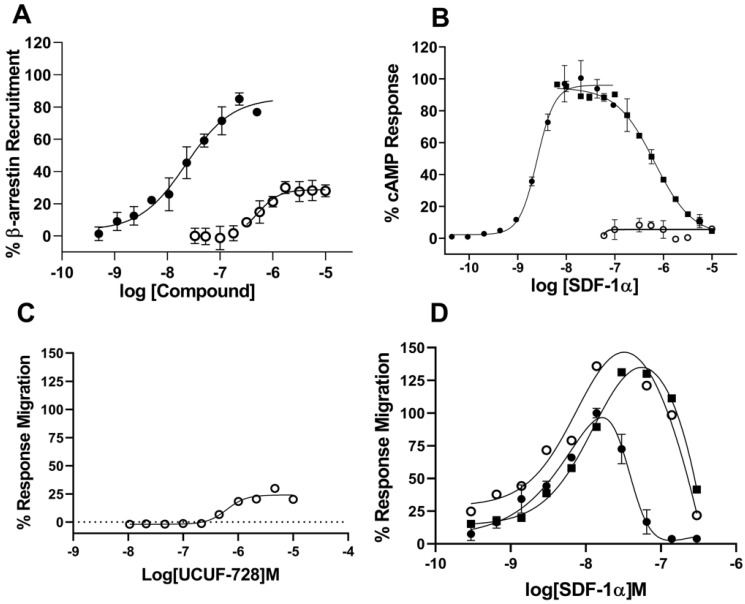
UCUF-728 is a positive modulator of b-arrestin signaling and cell migration. (**A**) UCUF-728 (open circles) is a dose-dependent partial activator of b-arrestin recruitment in CXCR4-bla U2OS cells compared to SDF-1a (closed circles). (**B**) SDF-1α dose-dependently inhibits forskolin-induced cAMP signaling (closed circles) in CHO-K1-CXCR4 cells, and this maximal response at 40 nM SDF-1α is inhibited by AMD3100 (closed squares). UCUF-728 (open circles) does not show dose-dependent activity in cAMP signaling. (**C**) UCUF-728 (open circles) is a dose-dependent partial activator of CEM-CCRF cell migration. (**D**) SDF-1a shows typical bell-shaped curve in CEM-CCRF cell migration (closed circles) and this response is modulated in the presence of 1 mM (closed squares) and 10 mM (open circles) UCUF-728. Error bars are +/− SD of triplicate determinations.

We further characterized UCUF-728 in 384 w assays using SDF-1α from a different source than was used in the high throughput screening assay. The new ligand was 3-fold more potent in our primary assay and was more economical (Supplemental Figure S5). Using this ligand source, UCUF-728 confirmed the activation of β-arrestin recruitment with EC_50_ = 0.5 µM and E_max_ = 30%, normalized to SDF-1α (Figure 2A).

The CXCR4 receptor is a G-protein coupled receptor (GPCR) coupled to the Gi class, whose primary role is inhibition of adenylate cyclase. We tested the ability of UCUF-728 to induce a Gi-mediated cAMP-inhibitory cellular response. SDF-1α inhibited forskolin-induced adenylate cyclase in CHO-K1 CXCR4 overexpressed cells and this response was dose-dependently blocked by AMD3100, a small bicyclam molecule that inhibits the binding of SDF-1α to CXCR4 [13] (Figure 2B). However, UCUF-728 did not inhibit forskolin-induced adenylate cyclase in the overexpressed cell line, indicating biased signaling for β-arrestin recruitment over cAMP signaling (Figure 2A,B).

SDF-1α stimulates chemotaxis in a high percentage of resting and active T lymphocytes [14], and the CXCR4 receptor is highly expressed in the CEM (Leukemia) cell line [15]. Thus, to confirm CXCR4 functional activity, we evaluated UCUF-728 in a 96 wells transwell migration assay in CEM human lymphoblast cells. The addition of lymphoblasts to the upper chamber and UCUF-728 to the lower channel induced migration with a similar EC_50_ and E_max_ as the B-arrestin recruitment activity (Figure 2A,C). The chemoattractant SDF-1α in the lower channel induced migration in the typical bell-shaped curve (Figure 2D) and this activity was blocked by the addition of AMD3100 (Supplemental Figure S6). Interestingly, the SDF-1α-mediated response was potentiated in the presence of saturating concentrations of UCUF-728 (Figure 2D). A partial agonist is expected to inhibit activity of a full agonist if both agonists bind to the same site on the receptor; our results suggested that UCUF-728 may not orthosterically bind to the CXCR4 receptor. To confirm this, we tested UCUF-728 in a CXCR4 Tag-lite binding assay and UCUF-728 did not displace SDF-1α binding, whereas, AMD3100 dose-dependently displaced SDF-1α with a potency comparable to the literature values [13] (Supplemental Figure S7). From the analog structure–activity relationship studies and characterization studies in the chemotaxis assay, cAMP signaling and CXCR4 binding assay, we identified UCUF-728 as a potent and functionally selective CXCR4 receptor modulator.

### 2.4. Ex Vivo Validation of the Leading Compound

**Figure 3:**
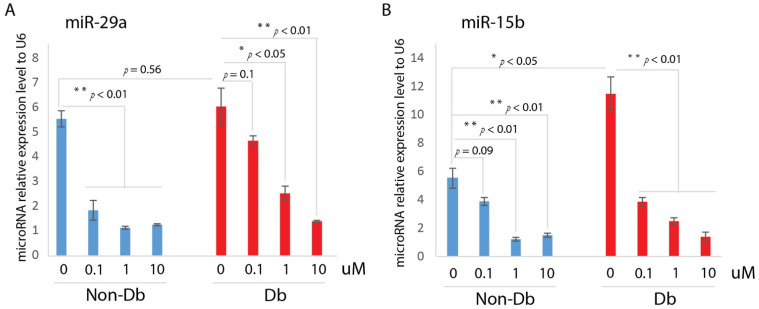
MiR-29a and miR-15b expression level was decreased with UCUF-728 treatment in Human dermal fibroblast. (**A**) Realtime qPCR (mean ± SD, *n* = 3 per group) analysis of miR-29a gene expression relative to internal control U6 in human diabetic dermal fibroblasts and non-diabetic dermal fibroblasts with UCUF-728 treated at dose of 1, 0.1, 1 and 10 uM. (**B**) Realtime qPCR (mean ± SD, *n* = 3 per group) analysis of miR-15b gene expression relative to internal control U6 in human diabetic dermal fibroblasts and non-diabetic dermal fibroblasts with UCUF-728 treated at dose of 1, 0.1, 1 and 10 uM. * *p* < 0.05; ** *p* < 0.01.

An additional secondary screen was performed to further validate the activity of UCUF-728 as a selective CXCR4 receptor modulator. Human diabetic and non-diabetic fibroblasts were cultured with increasing concentrations of the compound. The cells were incubated for 24 h and then total cellular RNA was isolated to examine the ability of the compound to correct the abnormal expression of miR-15b, and miR-29a. We have shown that human diabetic skin has an increased miR-15b expression at the baseline compared to non-diabetic skin [7]. We examined the effect of UCUF-728 on the human diabetic fibroblast expression of miR-15b and found that UCUF-728 treatment decreased the expression of miR-15b in human diabetic fibroblasts, similar to levels expressed by non-diabetic fibroblasts (Figure 3A). In preliminary studies, we have shown that the production of miR-29a is significantly upregulated in human diabetic skin at the baseline compared to non-diabetic skin [16]. Here, we found that human diabetic fibroblasts had increased the expression of miR-29a compared to non-diabetic fibroblasts, and that UCUF-728 resulted in a significant decrease in the miR-29a expression level (Figure 3B).

### 2.5. In Vivo Application of Leading Compound

**Figure 4:**
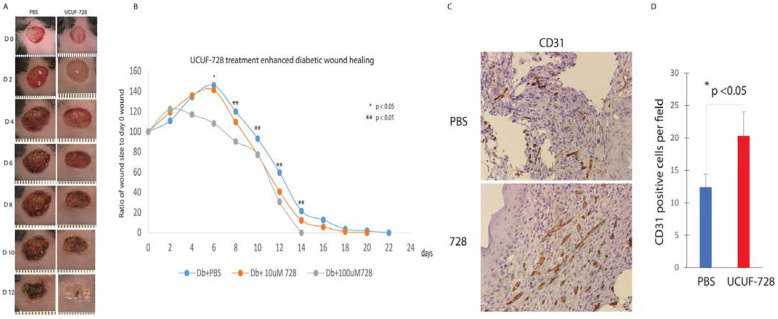
UCUF-728 enhances diabetic wound healing. (**A**) Representative photographs (days 0 to 12 post-wounding) of diabetic murine wounds treated with 0 (PBS), or 100 uM of UCUF-728 at the time of wounding. (**B**) Wound size change during the healing process of initial 8 mm wounds in Db mice treated by dermal injection of 10 uM UCUF-728 or PBS, *n* = 5 per group. (**C**) CD31+ staining of representative sections of diabetic wounds after 7 days of treatment with treated with PBS (*n* = 5) or 100 uM UCUF-728 (*n* = 5). (**D**) Quantitative analysis of number of vessels (CD31 staining) per 20× field. Comparison was performed between PBS and 100 uM UCUF-728 treated wounds. * *p* < 0.05; ** *p* < 0.01.

We examined the ability of our lead compound to improve diabetic wound healing in vivo. Full-thickness excisional 8 mm wounds were created in Db mice and were immediately treated with 10 µM UCUF-728 or PBS control. We monitored wound healing over the course of 22 days. Initial wound size was calculated immediately after wounding, and wound closure was assessed over time as the percentage of initial wound area. Figure 4A depicts the wound healing effect of UCUF-728 application on the excision wound model for sequential days following injury. By post-injury day 6, Db wounds treated with CAG 728 exhibited a decrease in the wound surface area compared to Db wounds treated with PBS. The time of full closure was 14 days compared to 22 days in Db wounds treated with PBS, indicating that UCUF-728 treatment in diabetic wounds significantly enhanced wound healing (Figure 4B). The effect of UCUF-728 treatment on angiogenesis was assessed using immunohistochemistry for the endothelial marker CD31. Representative photos of immunoperoxidase staining for CD31 at 7 days in diabetic wounds treated with PBS or UCUF-728 are shown in Figure 4C. Quantitative analysis of diabetic wounds treated with UCUF-728 demonstrated a significant increase in the number of vessels compared with PBS-treated diabetic wounds (Figure 4D).

## 3. Discussion

Secretion of the chemokine SDF-1α, with the subsequent activation of the CXCR4 receptor, is a critical component for effective wound healing [3,4,17]. It is a chemokine that promotes the recruitment of hematopoietic progenitor cells to areas of tissue injury [9,10]. The expression of SDF-1α is decreased in diabetic wounds, which may underlie the wound healing impairment depicted by an increased wound closure time, decreased granulation tissue, and a larger epithelial gap [3,4,5,6,7]. Reduced cellular migration resulting from decreased SDF-1a expression may contribute to observed healing impairment, as non-diabetic mouse wounds lacking lymphocytes recapitulated features of impaired wound healing, including preferential M1 polarization, increased basal ROS levels, and reduced angiogenesis [18]. Theses mechanistic insights highlight the utility of exploring novel therapeutics that can circumvent deficits in CXCR4 receptor activation to correct healing impairment.

Here, we developed a high-throughput β-arrestin recruitment assay to screen compounds for potential utility as CXCR4 receptor activators. The subsequent structure–activity relationship (SAR) studies identified a chemical scaffold that functions as a CXCR4 agonist (UCUF-728), and further activity was confirmed with in vitro and in vivo validation studies. The treatment of human diabetic fibroblasts with UCUF-728 resulted in the potent suppression of miR-15b expression, an outcome observed with the lowest concentration (0.1 μM). MiR-15b is a negative modulator of angiogenesis [19,20] that is upregulated in diabetic wounds during the early phase of healing (Xu et al., 2014). An increased expression of miR-15b is associated with a decreased expression of proangiogenic target genes, including vascular endothelial growth factor (VEGFα), hypoxia inducible factor (HIF-1α), and B-cell lymphoma 2 (BCL2) [7]. As previously demonstrated, the therapeutic suppression of miR-15b expression in diabetic wounds may contribute to accelerated wound closure by enhancing angiogenesis [7]. UCUF-728 treatment also reduced miR-29a expression in diabetic fibroblasts, but relative suppression was less potent than that observed of miR-15b. MiR-29a is upregulated in human and murine diabetic skin [16]. Evidence suggests that the dysregulation of miR-29a contributes to decreased collagen I protein content in diabetic wounds, leading to impaired biomechanical properties of skin that may underlie an increased susceptibility to injury [16].

In vivo, UCUF-728 treatment reduced wound closure time by 36%, which was observed. This data indicated that the topical application of UCUF-728 can accelerate diabetic wound healing, limiting the risks with systemic CXCR4 activation. Enhanced angiogenesis was also found in treated diabetic wounds. Together, these studies suggest that the activation of CXCR4 receptors with UCUF-728 accelerates wound healing by favoring the promotion of angiogenesis via the suppression of miR-15b. Mild reductions in miR-29a were observed, with near normalization of miR-29a expression to nondiabetic control levels (no treatment). This suggests that the promotion of the collagen I protein content, via the suppression of miR-29a, could also be contributing to UCUF-728-mediated wound repair.

This initial appraisal of the small molecule CXCR4 agonist, UCUF-728, demonstrates the utility of our approach for the development of therapies for impaired wound healing. Future studies will aim to optimize UCUF-728 lead for the suppression of miR-15b and miR-29a dysregulation, which may promote angiogenesis and collagen production, leading to more effective repairs of pathologic wound healing and reducing the probability of reinjury.

## 4. Materials and Methods

### 4.1. Reagents and Drug Treatment Protocol

All compounds were maintained as 10 mM DMSO stocks. To determine effects of compounds screened in cell-based assays, selected compounds were added to respective plates using the TECAN D300e digital dispenser. DMSO concentration was constant across all assay wells and did not exceed 0.5% in any cell-based assay. Unless otherwise indicated, reagents were purchased from Thermo Fisher Scientific (Waltham, MA, USA).

### 4.2. Cell Culture

CEM-CCRF cells (ATCC CCL-119) were cultured in growth media consisting of RPMI 1640 (Corning; Corning NY, USA) supplemented with 10% Fetal Bovine Serum (Corning; Corning, NY, USA), and 100 IU penicillin, 100 mg/mL streptomycin sulfate (Corning; Corning, NY, USA). Cells were maintained in suspension and subcultured twice weekly to maintain cell densities between 0.8 × 10^6^ cells/mL and 2.5 × 10^6^ cells/mL to maintain exponential growth phase. Cells were harvested for use in migration and calcium flux assays at 2.0 × 10^6^ cells/mL to 2.5 × 10^6^ cells/mL and resuspended in assay media to desired density for respective experiments. Tango™ CXCR4-bla U2OS Cells were maintained as a monolayer culture in McCoy’s 5a (Corning; Corning, NY, USA) supplemented with 10% dialyzed Fetal Bovine Serum, 0.1 mM MEM Non-essential amino acids solution, 25 mM HEPES solution (Corning; Corning, NY, USA), 1 mM Sodium Pyruvate, 200 ug/mL Zeocin selection reagent, 50 ug/mL Hygromycin B, and 100 ug/mL Geneticin. U2OS cells were routinely subcultured twice weekly by 0.25% Trypsin-EDTA treatment (Corning; Corning, NY, USA) and passaged to continue exponential growth phase for cell signaling assays. All cell cultures were maintained in cell culture incubators at 37 °C with 5% CO_2_. Human Dermal Fibroblasts were isolated from human skin biopsies according to the method [21]. Human dermal fibroblasts were cultured in full medium comprising Dulbecco’s modified eagle high-glucose (DMEM, Sigma-Aldrich, St. Louis, MO, USA) supplemented with 10% fetal bovine serum (FBS) and maintained at 37 °C in a humidified atmosphere containing 5% CO_2_. For further experiments, cells were seeded cultured for 12 h. Thereafter, cells were starved for 16 h and were stimulated with UCUF-728 at different dose.

### 4.3. B-Arrestin Recruitment

Compounds, SDF-1α (PeproTech; 30028A), and DMSO (Sigma Aldrich; St. Louis, MO, USA) normalization to 0.5% were dispensed using the TECAN D300e digital dispenser into a 384-well black wall clear-bottom microplate (Corning; Corning, NY, USA). Tango™ CXCR4-bla U2OS Cells were resuspended in assay media consisting of DMEM (Corning; Corning, NY, USA) supplemented with 1% dialyzed Fetal bovine serum, 0.1 mM MEM Non-essential amino acids solution, 25 mM HEPES solution (Corning; Corning, NY, USA), 1 mM Sodium Pyruvate and 100 IU penicillin, 100 mg/mL streptomycin sulfate (Corning; Corning, NY, USA) at a density of 3.0 × 10^5^ cells/mL. Cells suspension was dispensed at 30 μL per well into assay plate and incubated overnight in a cell culture incubator at 37 °C with 5% CO_2_. Following overnight incubation, LiveBlazer FRET B/G Loading Kit working reagent was prepared according to supplier guidelines and 6 μL of solution was dispensed into each well. Assay plate was covered and incubated in a dark place for two hours and Fluorescence Intensity was measured using the BMG LABTECH CLARIOstar Plus (BMG LABTECH Inc., Cary, NC, USA) according to the LiveBlazer FRET B/G Loading Kit excitation and emission measurement guidelines.

### 4.4. cAMP Signaling Assay

AMD3100, UCUF-728 and SDF-1a (PeproTech; Cranbury, NJ, USA) were dispensed in concentration-response mode using the TECAN D300e digital dispenser into white 384-OptiPlates (Perkin Elmer, Waltham, MA, USA). Forskolin at final concentration of 0.5 mM was added to all wells using the TECAN dispenser. Ten milliliters of PathHunter CHO-K1 CXCR4 cells (DiscoverX, Fremont, CA, USA) were added to compounds at 3000 cells/well, centrifuged at 1000 rpm for 30 s and incubated for 30 min at room temperature. DMSO (Sigma Aldrich, St. Louis, MO, USA) was normalized to 0.5%. Five nanoliters of 4X Eu-cAMP tracer working solution were added to all wells, followed by addition of 5 μL of 4X Ulight-anti-cAMP working solution to all wells. Plates were centrifuged at 1000 rpm for 1 min and incubated for 1 h at room temperature, then read using CLARIOstar Plus (BMG LABTECH Inc., Cary, NC, USA) Lance CAMP protocol.

### 4.5. Migration Assay

CEM-CCRF (ATCC CCL-119) were suspended in RPMI 1640 (Corning; 10-040-CV) at 5.0 × 10^5^ cells/mL and allowed to incubate in cell culture incubator at 37 °C with 5% CO_2_ for one hour. For agonist activity measurement, compounds, SDF-1α control ligand (PeproTech; 300-28A), and DMSO (Sigma Aldrich, St. Louis, MO, USA) normalized to 0.5% were dispensed into the compound receiver tray of Multi-Screen 96-well assay plate (Millipore Sigma, St. Louis, MO, USA) at desired concentration using the TECAN D300e digital dispenser. Following 1 h cell incubation and compound dispensing, 150 μL of RPMI 1640 supplemented with 2% Fetal Bovine Serum was added to each well of the compound receiver tray, and 50 μL of 5 × 10^5^ cells/mL cell suspension in RPMI were added to each well of the top filter plate. Plates were reassembled and placed in a cell culture incubator at 37 °C with 5% CO_2_ for three hours. After incubation, Multi-Screen plate was disassembled and 100 μL of solution was collected from compound receiver tray and transferred to 96 well luminescent plate and an equal volume of ATPLite 1step luminescence reagent (Perkin Elmer, Waltham, MA, USA) was added to each well. Luminescence was measured using the CLARIOstar Plus (BMG LABTECH Inc., Cary, NC, USA).

### 4.6. Binding Activity

Binding activity was measured using the Cisbio Tag-Lite Chemokine CXCR4 system (CisBio, Bedford, MA, USA). This assay was conducted using ready-to-assay Tag-Lite Chemokine CXCR4 labeled cells (C1TT1CXCR4), red fluorescent labeled CXCR4 ligand, and 1X Tag-Lite buffer prepared according to supplier guidelines. Compounds and AMD3100 were dispensed into a 384-well microplate (Greiner Bio, Frickenhausen Germany) using the TECAN D300e digital dispenser. CXCR4 Tag-lite cells were thawed and washed in 5 mL 1X Tag-Lite buffer, then re-suspended in 2.7 mL of 1X Tag-Lite buffer. After resuspension, 10 μL of cell suspension was dispensed into each assay well, followed by 5 μL of Tag-lite buffer and 5 μL of fluorescent ligand and allowed to incubate at room temperature in a dark place for three hours. Saturation binding and competition binding experiments were conducted on the same plate, with estimated Kd value of 12.5 nM fluorescent ligand for competition binding experiments. Following three-hour incubation, HTRF measurements were taken using the BMG LABTECH CLARIOstar Plus (BMG LABTECH Inc., Cary, NC, USA) with emission signals set to the Cisbio Tag-Lite Binding Assay supplier guidelines. HTRF ratio calculations completed according to supplier guidelines.

### 4.7. Animal Studies

All animal experiments were approved by the Institutional Animal Care and Use Committee at the University of Colorado Denver—Anschutz Medical Campus, and experimental protocols followed the guidelines described in the NIH Guide for the Care and Use of Laboratory Animals. In these experiments, we used 10-week-old, female, genetically diabetic C57BKS.Cg-m/Leprdb/J (Db) mice and heterozygous, non-diabetic (non-Db), age-matched female controls from the Jackson Laboratory (Bar Harbor, ME, USA). Mice were anesthetized with inhaled isoflurane. Each mouse was shaved and depilated before wounding. The dorsal skin was swabbed with alcohol and Betadine (Purdue Pharma, Stamford, CT, USA). Each mouse received a single, full-thickness dorsal wound (including panniculus carnosum) with an 8-mm punch biopsy (Miltex Inc., York, PA, USA). After wounding, a Hamilton syringe was used to deliver 50 μL of either 10 uM CAG1 or PBS, as a control. Ten nanoliters were injected intradermally at 12, 3, 6 and 9 o’clock and at the wound base. All wounds were dressed with Tegaderm (3M, St. Paul, MN, USA), which was subsequently removed on postoperative day 2. Postoperatively, the mice received a subcutaneous injection of an analgesic, Banamine (Schering-Plough Animal Health Corp., Union, NJ, USA). A full-thickness skin sample, centered on the wound, was harvested 3 and 7 days after surgery (*n* = 5 per timepoint).

### 4.8. Real Time Quantitative PCR

Total RNA was extracted with TRIzol reagent (Invitrogen, Carlsbad, CA, USA) according to the manufacturer’s established protocol. RNA was converted into cDNA using the SuperScript First-Strand Synthesis System (Invitrogen, Life Technologies). Primers and probes for mouse miR-15b and miR-29a were acquired from Applied Biosystems TaqMan gene expression assay (Applied Biosystems, Foster City, CA, USA). Quantitative PCR was performed on a BIO-RAD CFX96 according to the manufacturer’s instructions. Quantitative values of genes of interest are normalized based on U6. Samples (*n* = 5 per group) were amplified in triplicate and results were averaged for each individual sample. The ΔΔCT method was used to calculate relative gene expression. Results are reported as mean ± SD.

### 4.9. Statistical Analysis

Results are expressed as mean ± SD for 3 to 5 independent experiments. Statistically significant differences in gene expression between two groups were assessed by Student’s *t*-test, ANOVA with an appropriate post hoc test was to be used for multiple comparisons. *p* < 0.05 was considered to be statistically significant. All concentration response curves were analyzed to determine EC_50_ and E_max_ using the following equation:

$$y=\frac{100}{1+10^{log\left(EC50-x\right)}\;x\;Hillslope}$$

## Figures and Tables

**Table 1:** SAR of analogs of the 5-aryl-2-cyclopropyloxazole series in the primary CXCR4 b-arrestin recruitment assay.

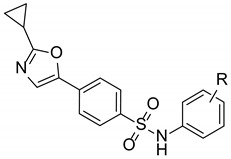			
Entry	R	CXCR4 β-Arrestin EC_50_ (mM)	CXCR4 β-Arrestin E_max_ (%)
**1**	H	>80	
**2**	2-Me	5.9	27
**3**	2-Et	2.6	38
**4**	2-OMe	6.1	45
**5**	2-OEt	1.0	46
**6**	2-Cl	22	35
**7**	2-F	>80	
**8**	3-OMe	>80	
**9**	4-OEt	44.3	38
**10**	2,3-dimethyl	1.9	40
**11**	3,4-dimethyl	>10	
**12**	2,4-dimethyl	3.4	38
**13**	2,5-dimethyl	0.7	42
**14**	2,6-dimethyl	0.6	45
**15**	3,5-dimethyl	3.1	39
**16**	2-OMe, 5-Cl	1.65	47
**17**	2-OMe, 5-F	7.3	42
**18**	2-OMe, 5-Me	0.9	48
**19 (UCUF-728)**	2,5-dimethoxy	1.1	60
**20**	3,4-dimethoxy	>80	
**21**	3,5-dimethoxy	5.6	48

## Data Availability

No datasets were generated or analyzed during the current study.

## Supplementary Materials

The following supporting information can be downloaded at https://www.mdpi.com/article/10.3390/ijms23042196/s1.
