# Supplementary material for: Discovery of Small Molecule Activators of Chemokine Receptor CXCR4 That Improve Diabetic Wound Healing
Source: Int J Mol Sci. 2022 Feb 16;23(4):2196. doi: 10.3390/ijms23042196 (PMC8879702; doi:10.3390/ijms23042196)
Supplement: Supplementary file 1 [file ijms-23-02196-s001.zip › ijms-1582355-supplementary.pdf]

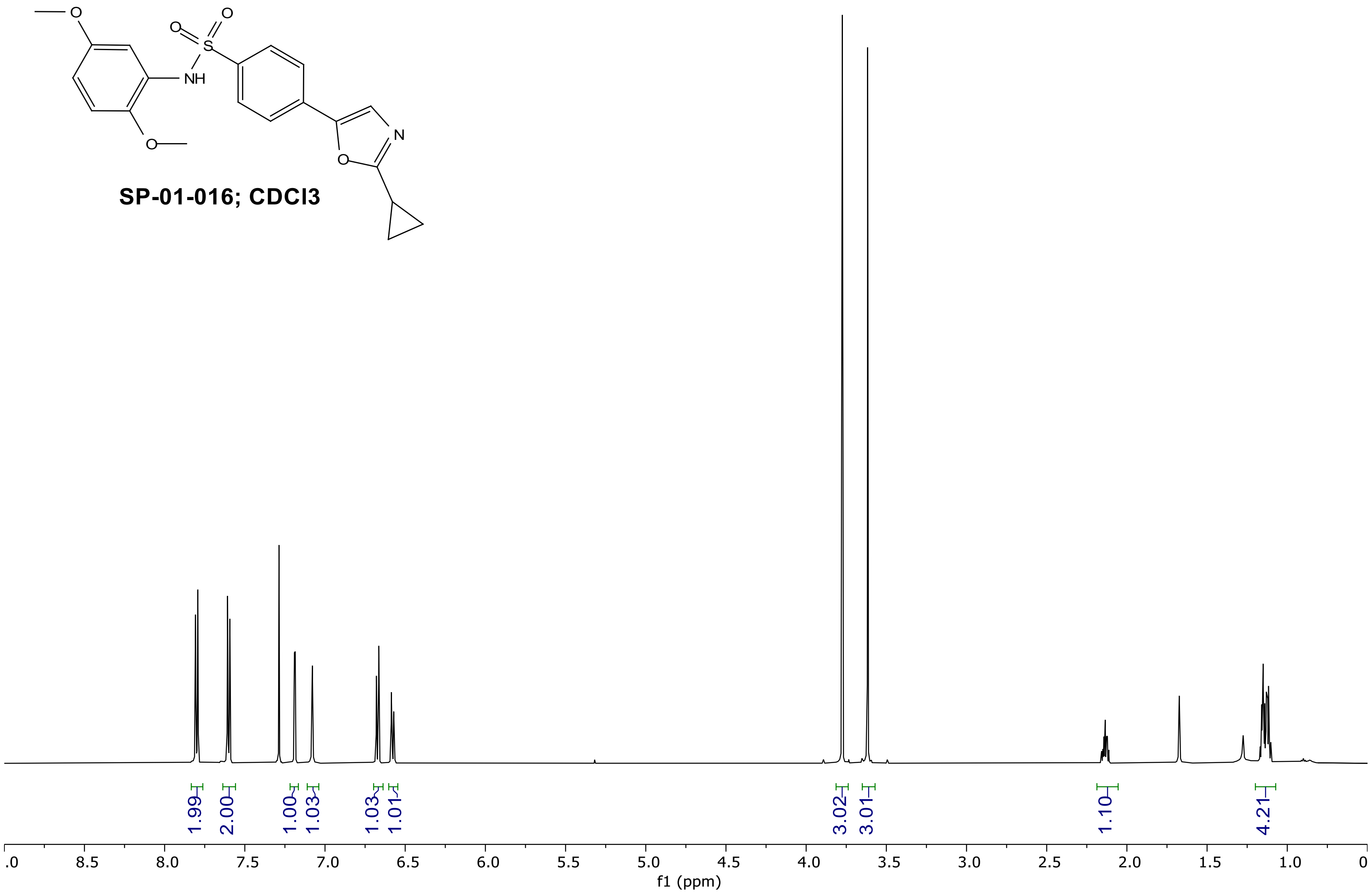

Figure S1. Protein NMR Spectra of UCUF-728. ^{1}H NMR (600 MHz, $CDCl_3$) δ 7.80 (d, *J* = 8.6 Hz, 2H), 7.60 (d, *J* = 8.6 Hz, 2H), 7.19 (d, *J* = 3.0 Hz, 1H), 7.08 (s, 1H), 6.67 (d, *J* = 8.9 Hz, 1H), 6.58 (dd, *J* = 8.9, 3.0 Hz, 1H), 3.77 (s, 3H), 3.62 (s, 3H), 2.18 – 2.06 (m, 1H), 1.19 – 1.06 (m, 4H).

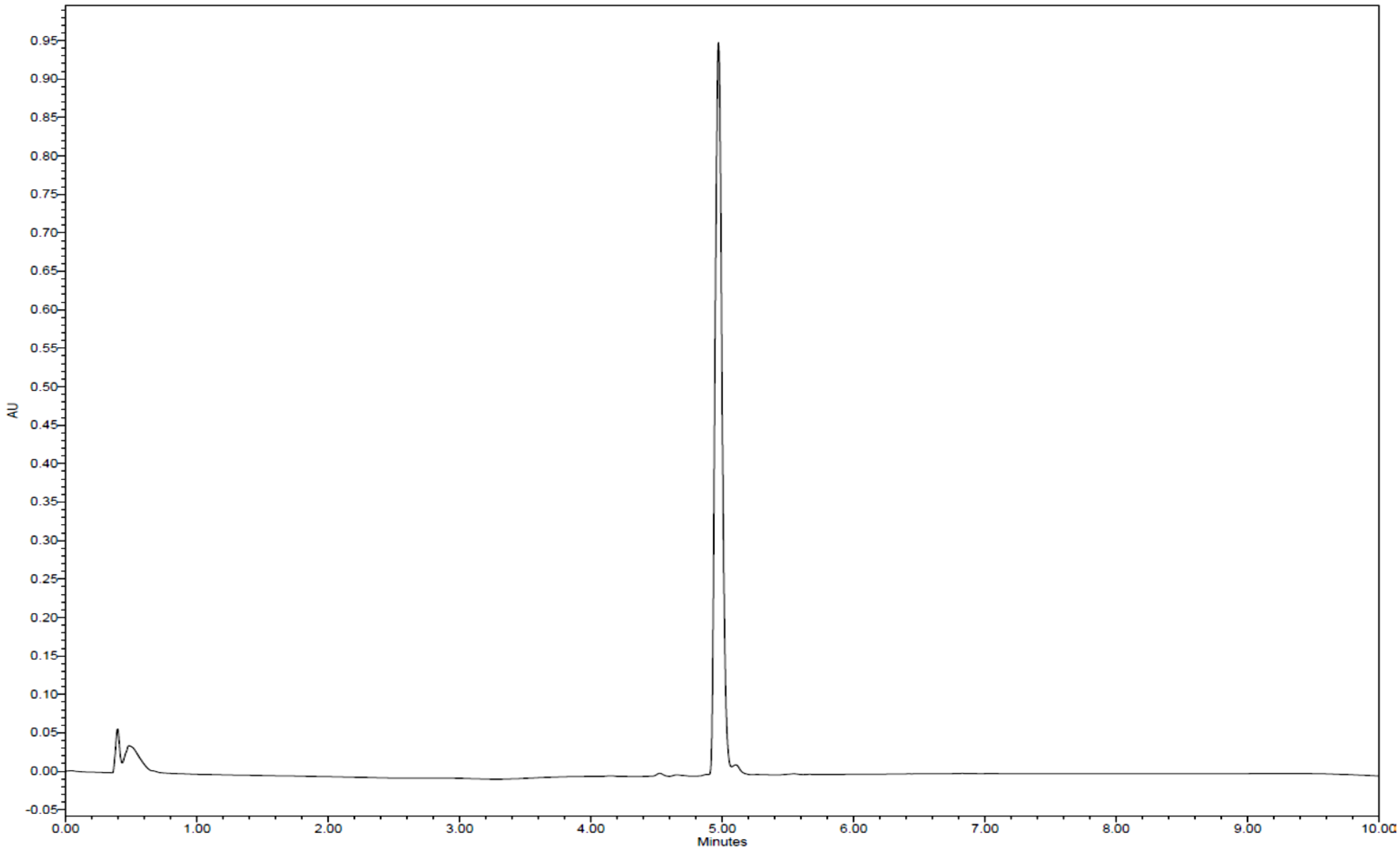

Figure S2. Liquid chromatography Spectra of UCUF-728 at 254 nm.

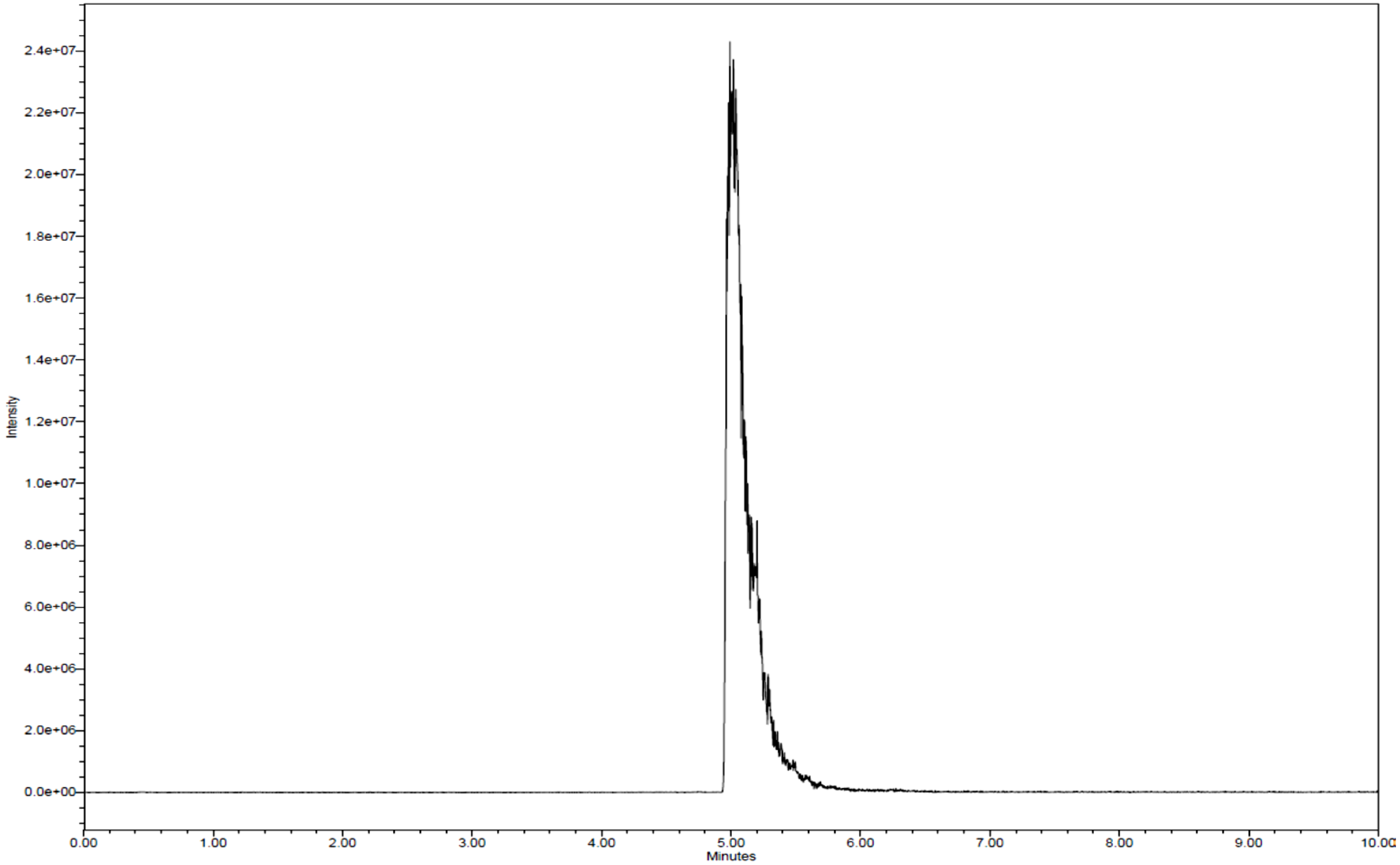

Figure S3. Mass Spectrometry Chromatogram of UCUF-728.

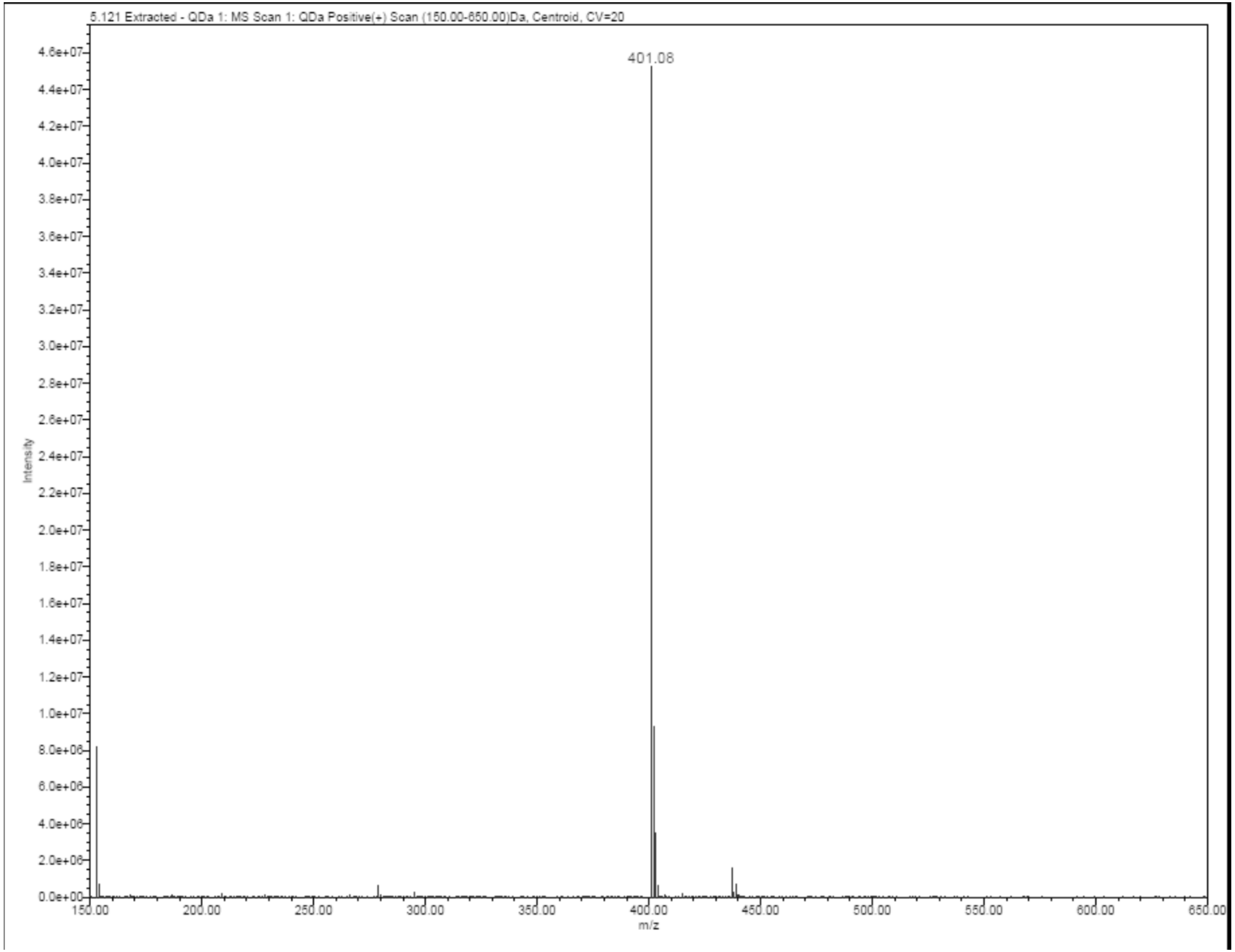

Figure S4. Mass Spectrometry of UCUF-728. Chemical Formula: $C_{20}H_{20}N_2O_5S$
Calculated [M+H]: 401.12

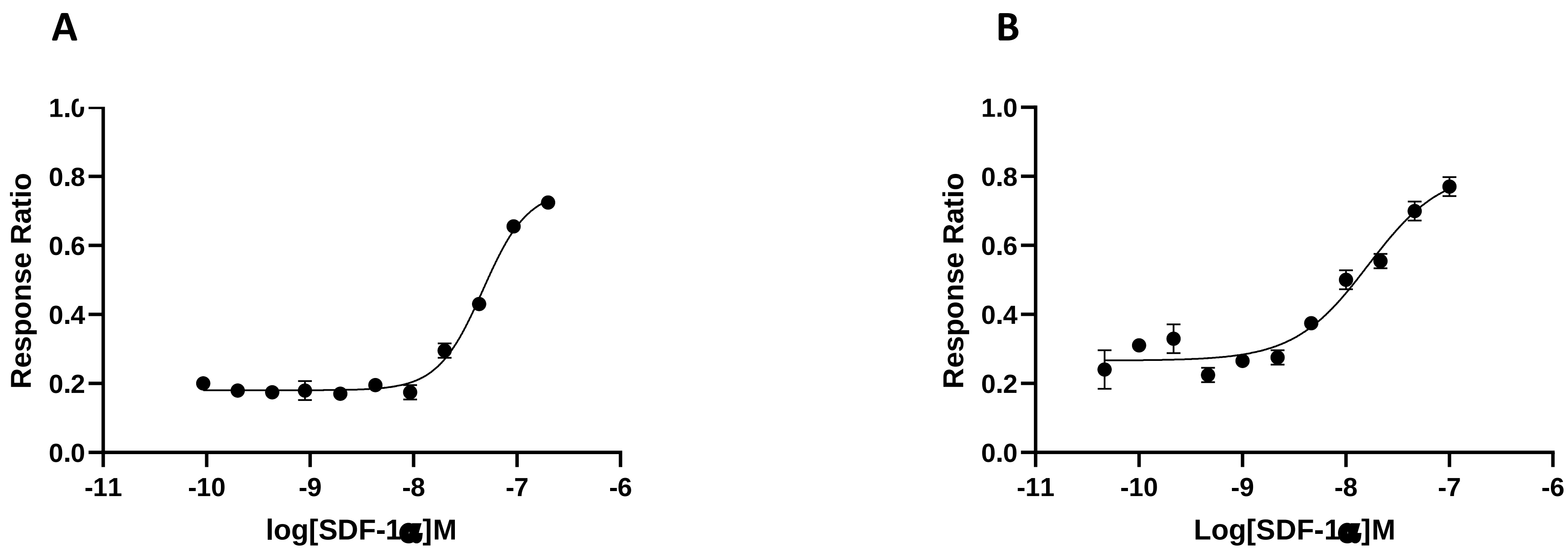

Figure S5. Dose-dependent β-arrestin recruitment in U2OS-CXCR4*bla* cells of SDF-1α purchased from A) R&D Systems (Log EC_{50} = -7.33 ± 0.06) and B) Peprotech (Log EC_{50} = -7.78 ± 0.05).

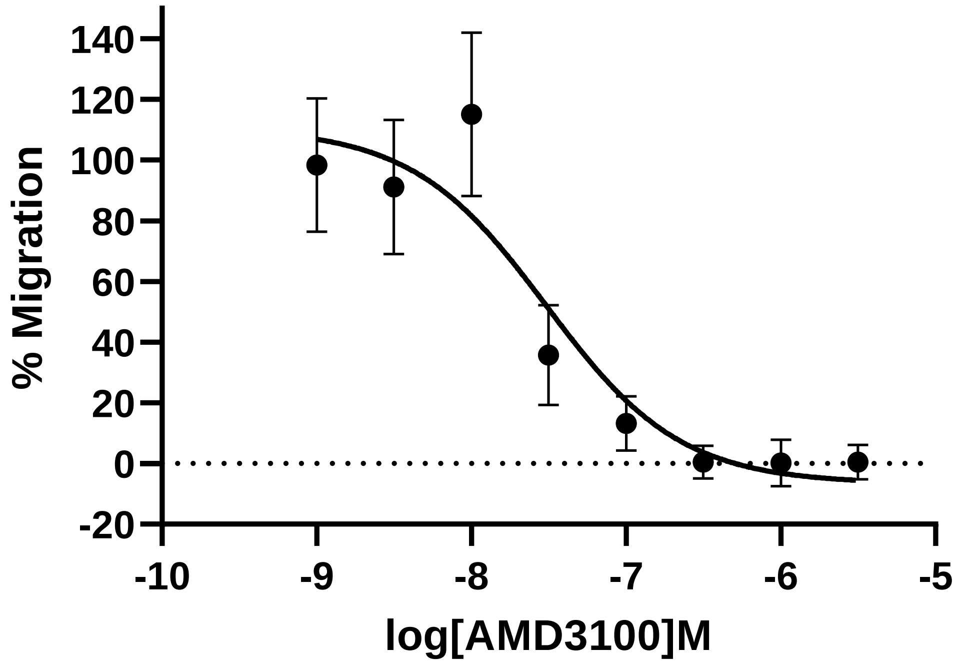

Figure S6. Percent Migration of Lymphoblasts Antagonized by AMD 3100. AMD3100 dose-dependently inhibited migration in CEM lymphoblasts with $logIC_{50}$ = -7.5 ± 0.6. Data is normalized to media only (low control) and 6nM SDF-1α (high control). Data is average of duplicate experiments performed n triplicate.

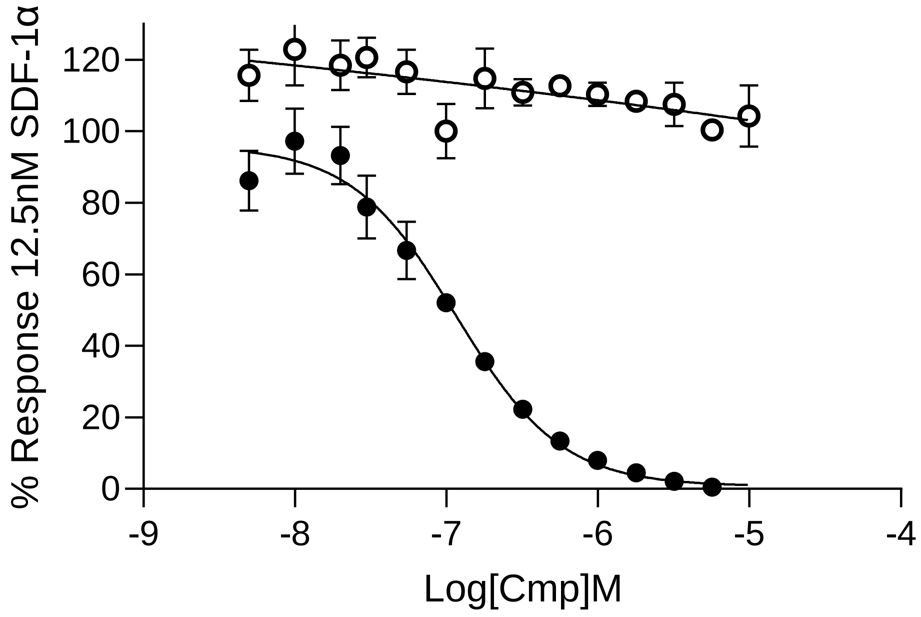

Figure S7. Antagonism of SDF-1α binding to CXCR4 Receptor. SDF-1α binding was validated using the Tag-Lite CXCR4 system (CisBio) according to manufacturer protocol. AMD3100 dose-dependently displaced SDF-1α saturation binding (closed circles) and UCUF-728 did not displace SDF-1α saturation binding (open circles).
